# Incident Subjective Cognitive Decline Does Not Predict Mortality in the Elderly – Results from the Longitudinal German Study on Ageing, Cognition, and Dementia (AgeCoDe)

**DOI:** 10.1371/journal.pone.0147050

**Published:** 2016-01-14

**Authors:** Susanne Roehr, Tobias Luck, Kathrin Heser, Angela Fuchs, Annette Ernst, Birgitt Wiese, Jochen Werle, Horst Bickel, Christian Brettschneider, Alexander Koppara, Michael Pentzek, Carolin Lange, Jana Prokein, Siegfried Weyerer, Edelgard Mösch, Hans-Helmut König, Wolfgang Maier, Martin Scherer, Frank Jessen, Steffi G. Riedel-Heller, AgeCoDe Study Group

**Affiliations:** 1 Institute of Social Medicine, Occupational Health and Public Health (ISAP), University of Leipzig, Leipzig, Germany; 2 LIFE–Leipzig Research Center for Civilization Diseases, University of Leipzig, Leipzig, Germany; 3 Department of Psychiatry, University of Bonn, Bonn, Germany; 4 Institute of General Practice, Medical Faculty, Heinrich-Heine-University Düsseldorf, Düsseldorf, Germany; 5 Department of Primary Medical Care, Center for Psychosocial Medicine, University Medical Center Hamburg-Eppendorf, Hamburg-Eppendorf, Germany; 6 Work Group Medical Statistics and IT-Infrastructure, Institute for General Practice, Hannover Medical School, Hannover, Germany; 7 Central Institute of Mental Health, Medical Faculty, Mannheim/Heidelberg University, Mannheim, Germany; 8 Department of Psychiatry, Klinikum rechts der Isar, Technical University of Munich, Munich, Germany; 9 Department of Health Economics and Health Services Research, Hamburg Center for Health Economics, University Medical Center Hamburg-Eppendorf, Hamburg, Germany; 10 German Center for Neurodegenerative Diseases, DZNE, Bonn, Germany; 11 Department of Psychiatry, University of Cologne, Cologne, Germany; University of Naples Federico II, ITALY

## Abstract

**Objective:**

Subjective cognitive decline (SCD) might represent the first symptomatic representation of Alzheimer’s disease (AD), which is associated with increased mortality. Only few studies, however, have analyzed the association of SCD and mortality, and if so, based on prevalent cases. Thus, we investigated incident SCD in memory and mortality.

**Methods:**

Data were derived from the German AgeCoDe study, a prospective longitudinal study on the epidemiology of mild cognitive impairment (MCI) and dementia in primary care patients over 75 years covering an observation period of 7.5 years. We used univariate and multivariate Cox regression analyses to examine the relationship of SCD and mortality. Further, we estimated survival times by the Kaplan Meier method and case-fatality rates with regard to SCD.

**Results:**

Among 971 individuals without objective cognitive impairment, 233 (24.0%) incidentally expressed SCD at follow-up I. Incident SCD was not significantly associated with increased mortality in the univariate (HR = 1.0, 95% confidence interval = 0.8–1.3, *p* = .90) as well as in the multivariate analysis (HR = 0.9, 95% confidence interval = 0.7–1.2, *p* = .40). The same applied for SCD in relation to concerns. Mean survival time with SCD was 8.0 years (*SD* = 0.1) after onset.

**Conclusion:**

Incident SCD in memory in individuals with unimpaired cognitive performance does not predict mortality. The main reason might be that SCD does not ultimately lead into future cognitive decline in any case. However, as prevalence studies suggest, subjectively perceived decline in non-memory cognitive domains might be associated with increased mortality. Future studies may address mortality in such other cognitive domains of SCD in incident cases.

## Introduction

Current research provides growing evidence that subjective cognitive decline (SCD) may carry an increased risk of future cognitive decline and Alzheimer’s disease (AD) at a point when performance on cognitive tests still is objectively normal [1–14].

Therefore, SCD might be helpful in the very early detection of neurodegenerative processes, and may hold value for future prevention approaches. This is moreover important since dementia, with AD as the most common type, is associated with increased mortality [15–18]. Following the suggestion, that SCD might represent the earliest symptomatic manifestation of AD, and dementia being associated with increased mortality, the assumption arises, that mortality risk may already be increased when SCD is firstly expressed.

Previous studies addressing the association of SCD and mortality yielded inconsistent results: some studies found SCD not being related to increased mortality [19–22], others reported increased mortality only in certain features of SCD [23–25] while again others did find increased mortality risk in SCD [26,27].

There are several potential explanations for the inconclusive picture. First, until recently there was a lack of a consensus definition for SCD which might have led to a mingling of cognitive complaints related to such various reasons as psychiatric diseases, e.g. depression and anxiety, substance abuse, or medication intake. Further, there is a variety of strategies concerning the assessment of SCD from one question to standardized questionnaires applied to various populations in different research environments.

Another main reason for the preliminary results might be the investigation of prevalent cases in previous studies. In prevalent cases, information on the onset of SCD is lost which is likely to lead to length bias and, hence, less precise outcomes on mortality [28]. Calculations from SCD onset in incident cases, by contrast, may provide more pronounced estimates on mortality.

### Aims of the study

Thus, we sought to investigate the association of incident SCD from their onset and mortality in the elderly, a population in which SCD is very frequent and which is at high risk for AD. We specifically examined SCD in memory, as one particular type of SCD, as research evidence on the association of memory function with preclinical AD may be strongest at present.

According to the SCD research criteria by Jessen et al. [4], we defined SCD as the awareness of a self-experienced decline in memory compared to a previous better status which was not related to an actual event or underlying condition that could have explained the decline. Furthermore, we differentiated SCD in relation to concerns regarding mortality risk. Finally, we calculated estimates on survival time and case-fatality rates in regard to SCD and in relation to concerns.

## Material and Methods

### Study design and sample

Data were derived from the German Study on Ageing, Cognition, and Dementia in Primary Care Patients (AgeCoDe), a prospective longitudinal study on the early detection of mild cognitive impairment and dementia in general practice. The AgeCoDe study was conducted at six German cities (Bonn, Duesseldorf, Hamburg, Leipzig, Mannheim, and Munich). Recruitment of the participants by 138 general practitioners (GP) took place between January 1, 2003 and November 30, 2004. To be included in the study, individuals had to be at least 75 years old, without dementia as assessed by the GP, and had to have at least one contact with the GP within the last year. Exclusion criteria comprised GP consultations at home only, nursing home residence, severe illness with an expected fatal outcome within three months, German language insufficiency, deaf- or blindness, and the inability to provide informed consent.

**Fig 1:**
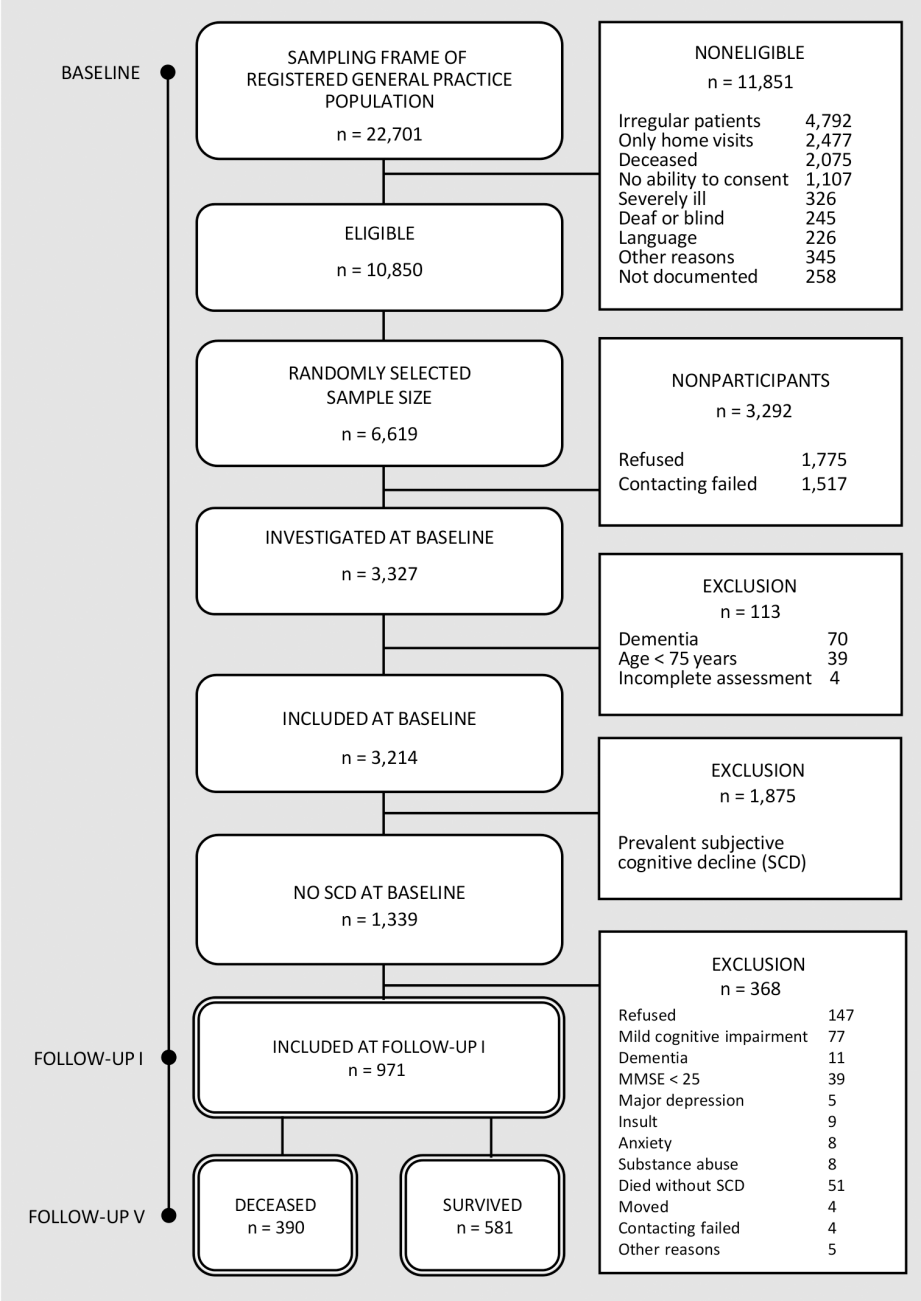
Sample attrition and sample.

At baseline, 3,327 GP patients were investigated, of which 2,326 (69.9%) were women. Of the total sample, 113 (3.4%) individuals had to be excluded because of prevalent dementia (*n* = 70, 2.1%), a younger age than 75 years (*n* = 39, 1.2%), and incomplete assessments (*n* = 4, 0.1%). After all, 3,214 individuals constituted the AgeCoDe study population (Fig 1). More study details have been previously described elsewhere [29].

### Ethics

The AgeCoDe-study was conducted in accordance with the Declaration of Helsinki [30] and was approved by the local ethic committees of all participating centers (file reference numbers: Ethics Commission of the Medical Association Hamburg: OB / 08 / 02 & 2817/2007; Ethics Commission of the University of Bonn: 050/02 & 174/02 for E 3.2 & 258/07; Medical Ethics Commission II, University of Heidelberg at the University Medical Center of Mannheim: 0226.4 & 2002 2007-253E-MA; Ethics Commission at the Medical Center of the University of Leipzig: 143/2002 & 309/2007; Ethics Commission of the Medical Faculty of the Heinrich-Heine-University Düsseldorf: 2079/2002 & 2999/2008; Ethics Committee of the TUM School of Medicine, Munich: 713/02 & 713/02 E). Patients and/or their proxies provided written informed consent.

### Data collection and assessment procedures

Data were analyzed up to follow-up 5 and were collected between January 1, 2003 (begin of baseline) and October 29, 2012 (end of follow-up 5) comprising an observation period of 7.5 years. Follow-up assessments took place on average every 1.5 years. Trained psychologists and physicians visited GP patients at home and conducted structured clinical interviews.

The main instrument of the AgeCoDe-study was the Structured Interview for the Diagnosis of Dementia of Alzheimer Type, Multi-infarct Dementia, and Dementia of other Aetiology according to DSM-III-R, DSM-IV, and ICD-10 (SIDAM) [31]. Among others, the SIDAM includes a cognitive test battery with 55 items that cover four main domains of cognitive functioning: orientation, memory, intellectual abilities, and higher cortical functioning. The 55 items include the 30 items of the Mini Mental State Examination (MMSE) [32,33].

SCD in memory was evaluated by asking the subjects “Do you feel as if your memory is becoming worse?” (yes/no/I don’t know) prior to cognitive testing. In case of a positive answer, we specified if SCD was related to concerns by asking “Does this worry you?” (yes/no/I don’t know).

Depressive symptoms were assessed with the short version of the Geriatric Depression Scale (GDS) [34] and impairment in instrumental activities of daily living (IADL) with the Lawton-and-Brody IADL scale [35].

Information on smoking and alcohol consumption was gathered based on a standardized questionnaire. Regarding smoking status, we grouped participants into non-smokers, former and current smokers. Alcohol consumption was assessed by rating the amount of alcohol consumed on a regular basis in categories from no drinking to alcohol dependence according to guidelines of the World Health Organisation [36].

GPs provided information on the presence of comorbidity through completing standardized questionnaires at each study wave and took blood samples at baseline for genetic analyses.

Due to its potential as a genetic risk factor for AD, carrying of Apolipoprotein E epsilon 4 allele (apoE4) was assessed according to standard procedures [37]. Subjects were grouped into apoE4 carriers and non-apoE4 carriers.

If participants could not be reached by mail or phone at follow-up, a contact person (commonly spouse, children, or other relatives) was phoned and interviewed. Death dates were obtained from the contact persons, GPs, or the local residents’ registration office that registers all Germans by law.

### Definition of cases

#### SCD

SCD cases were considered those individuals who expressed memory complaints besides age- and education-specific normal performance on standard objective cognitive tests. Consequently, we excluded all cases with a coincident diagnosis of dementia, MCI and cognitive functioning on the MMSE worse than 25 points. Moreover, we excluded cases in which SCD could be explained through a psychiatric, neurological and medical disorder or substance abuse as rather functional memory impairment of the underlying condition. Specifically, individuals were excluded in case of a major depression (GDS > 9), anxiety disorders, substance abuse and stroke (as reported by the GPs) until follow-up I.

Incident cases were considered those individuals who expressed SCD at follow-up I while not stating SCD at baseline. Prevalent cases of SCD at baseline were excluded.

#### MCI

Diagnosis of MCI was based on current consensus criteria [38] that comprise: absence of dementia according to DSM-IV, at most minimal impairment in instrumental functions as assessed by the SIDAM-ADL scale (maximum of one impairment) and evidence of cognitive decline in self- or informant report and in objective cognitive tests (i.e. subjects and/or their proxies reported SCD, and test performance on one or more main domains of cognitive functioning as assessed by the SIDAM was one standard deviation below the age- and education specific norms).

#### Dementia

Dementia at baseline and at follow-up waves was diagnosed by interviewers and experienced geriatricians in a consensus conference according to the DSM-IV criteria [31], which are implemented as a standardized diagnostic algorithm in the SIDAM. If SIDAM results were unavailable, dementia diagnosis was based on a cut off score of ≥ 4 on the Global Deterioration Scale [39] and a total score of ≥ 9 on the Blessed Dementia Rating [40] subscales as judged by proxies.

### Statistical analyses

Group differences in socio-demographic and health characteristics at onset of incident SCD were analyzed in respect to mortality status by applying Mann-Whitney *U* tests for continuous variables and *χ*^*2*^ tests for categorical variables.

Onset of incident SCD was assumed at the midway of baseline and follow-up 1, whereby the latter was the point of first expression. For individuals without SCD at follow-up 1, the beginning of the observation period was calculated accordingly. For potentially time dependent variables, we chose data from the interview closest to incident SCD onset or begin of the observation period in individuals without SCD, respectively.

Mortality was defined as the time interval from date of SCD onset or comparable begin of observation in cases without SCD to date of death in years. Individuals who were still alive by the end of follow-up 5 were treated as censored data. Attained age was measured as date of birth to date of death or date of last contact for survivors. Person-years of observation were defined as time between SCD onset/begin of observation period in individuals without SCD and death or last day of contact. We calculated mortality by dividing the number of deaths by person-years of observation (case-fatality rates).

We calculated univariate and multivariable Cox proportional hazards regression models with hazard ratios (HR) and Wald 95%-confidence intervals (CI) to assess the effect of a) SCD and b) SCD in relation to concerns on mortality. The multivariate Cox models were adjusted for potential confounders comprising age, cognitive functioning (MMSE) and depressive symptoms (GDS) as continuous variables and gender, education (categorized into low, middle and high according to the CASMIN criteria [41]), living situation, marital status, impairment in IADL, smoking, drinking, apoE4, subsequent dementia and comorbidity as categorical variables. Comorbid conditions included diabetes mellitus, hypertension, cardiac arrhythmias, coronary heart disease, myocardial infarction, stenosis and transient ischaemic attack (TIA). The proportional hazards assumption for all applied models was tested calculating Schoenfeld residuals [42].

Kaplan-Meier survival analyses were applied to determine the survival times with regard to SCD and to SCD in relation to concerns. A Log-rank test was performed to assess the survival difference between individuals with and without SCD and SCD in relation to concerns.

All statistical analyses were executed with Stata/SE, version 13.0 (StataCorp LP, College Station, Texas/USA), and SPSS, version 20.0 (IBM, Armonk, New York/USA). The significance level was set at *α* = 0.05 for all calculations.

## Results

### Descriptive characteristics

Of 3,214 AgeCoDe cohort subjects at baseline, 1,875 (58.3%) cases of prevalent SCD were excluded from the analysis. Among the remaining 1,339 subjects (41.7%), 971 cases were included (72.5%) at follow-up I and more 368 (27.5%) cases were excluded (Fig 1). Excluded individuals were significantly older (*M* = 79.9, *SD* = 3.7 vs. *M* = 79.3, *SD* = 3.4; *U* = 1007727.0, *p* < 01), more frequently male (35.6% vs. 32.0%; *χ*^*2*^(1, 3214) = 3.87, *p* < .05), and had lower MMSE scores than study participants (*M* = 27.3, *SD* = 2.0 vs. *M* = 27.9, *SD* = 1.5; *U* = 917553.0, *p* < .001), but did not differ in terms of education (*χ*^*2*^(2, 3214) = 4.08, *p* = .13).

Regarding the 971 included cases, 233 (24.0%) incidentally expressed SCD at follow-up I. At incident SCD onset, individuals were *M* = 80.7 (*SD* = 3.4, range = 75.6–91.8) years old, and individuals without SCD were *M* = 80.4 (*SD* = 3.3, range = 77.4–98.6) years old (*U* = 82456.5, *p* = .35). In 41 cases (17.6%) SCD was accompanied by related concerns. The total observation time cumulated in 7,144.4 person-years. During a mean follow-up time of *M* = 7.4 (*SD* = 2.1) years, 415 (42.7%) individuals completed all follow-ups, 166 (17.1%) individuals were lost to follow-up, and 390 (40.2%) individuals had died. Among the 233 individuals with SCD, 96 (41.2%) died, and among those 738 individuals without SCD, 294 (39.8%) died (*χ*^*2*^(1, 971) = 0.14, *p* = .71). Individuals who were lost to follow-up were significantly younger (*M* = 78.6 years, *SD* = 3.1 years vs. *M* = 79.5, *SD* = 3.4 years; *U* = 57267.5, *p* < .01) and less educated (*χ*^*2*^(2, 971) = 13.00, *p* < .01) than individuals who had died or remained in the study until censoring, but they did not differ in terms of cognitive functioning (MMSE: *M* = 27.8, *SD* = 1.6 vs. *M* = 27.9, *SD* = 1.4; *U* = 64073.0, *p* = .40) and gender (*χ*^*2*^(1, 971) = 1.72, *p* = .19).

Table 1 outlines socio-demographic and health characteristics of the study sample by SCD status. Individuals with SCD showed significantly higher depressive symptoms (*U* = 74714.5, *p* < .01) and suffered more often from a transient ischaemic attack (TIA) (*χ*^*2*^(1, 967) = 3.87, *p* < .05) than individuals without SCD. Groups did not differ in any other characteristic.

**Table 1:** Socio-demographic and health characteristics of the study sample by status of subjective cognitive decline (SCD) (n = 971).

Variables^§^		Total sample (n = 971)	Subjects with SCD (n = 233)	Subjects without SCD (n = 738)	*P* value
Age at onset, *M* (*SD*)		80.50 (3.35)	80.70 (3.44)	80.44 (3.32)	.35
Gender, *n* (*%*)					
	Female	660 (68.0)	148 (63.5)	512 (69.4)	
	Male	311 (32.0)	85 (36.5)	226 (30.6)	.10
Education, *n* (*%*)					
	High	88 (9.1)	24 (10.3)	64 (8.7)	
	Middle	265 (27.3)	50 (21.5)	215 (29.1)	
	Low	618 (63.6)	159 (68.2)	459 (62.2)	.07
Marital status, *n* (*%*)					
	Single	64 (6.6)	14 (6.0)	50 (6.8)	
	Married/cohabiting	379 (39.0)	90 (38.6)	289 (39.2)	
	Divorced	62 (6.4)	14 (6.0)	48 (6.5)	
	Widowed	466 (48.0)	115 (49.4)	351 (47.6)	.95
Living situation, *n* (%)					
	Private household, alone	517 (53.2)	115 (49.4)	402 (54.5)	
	Private household, with relatives	434 (44.7)	113(48.5)	321 (43.5)	
	Residential care	20 (2.1)	5 (2.1)	15 (2.0)	.39
Cognitive functioning^$^, *M* (*SD*)		27.90 (1.45)	27.82 (1.57)	27.93 (1.42)	.57
Impairment in instrumental activities of daily living^†^, n (*%*)		53 (5.5)	16 (6.9)	37 (5.0)	.28
**Depressive symptoms**^**‡**^**, *M* (*SD*)**		**1.62 (1.84)**	**1.94 (1.97)**	**1.51 (1.78)**	**< .01**
Worry related to SCD, *n* (*%*)		N/A	41 (17.6)	N/A	N/A
Feelings of worse memory performance than same aged individuals, *n* (*%*)		10 (1.0)	5 (2.1)	5 (0.7)	.05
Comorbid conditions, *n* (*%*)					
	Diabetes mellitus	218 (22.5)	55 (23.7)	163 (22.2)	.63
	Hypertension	696 (71.7)	169 (72.8)	527 (71.7)	.74
	Cardiac arrhythmias	286 (29.5)	74 (31.9)	212 (28.8)	.37
	Coronary heart disease	311 (32.0)	85 (36.6)	226 (30.7)	.09
	Myocardial infarction	81 (8.3)	18 (7.8)	63 (8.6)	.70
	Peripheral artery occlusive disease (PAOD)	76 (7.8)	19 (8.2)	57 (7.8)	.83
	Stenosis (of afferent brain vessels)	22 (2.3)	5 (2.2)	17 (2.3)	.89
	**Transient ischaemic attack (TIA)**	**44 (4.5)**	**16 (6.9)**	**28 (3.8)**	**< .05**
Smoking, *n* (*%*)					
	Non-smoker	515 (53.0)	118 (50.6)	397 (53.8)	
	Former smoker	367 (37.8)	99 (42.5)	268 (36.3)	
	Current smoker	89 (9.2)	16 (6.9)	73 (9.9)	.14
Alcohol consumption^¥^, *n* (*%*)					
	No drinking	497 (51.2)	111 (47.6)	386 (52.6)	
	Normal drinking	437 (45.0)	113 (48.5)	324 (44.1)	
	Risky drinking	33 (3.4)	9 (3.9)	24 (3.3)	.41
apoE4, *n* (*%*)					
	No apoE4	765 (81.8)	184 (81.1)	581 (82.1)	
	apoE4	170 (18.2)	43 (18.9)	127 (17.9)	.73
Subsequent incident dementia, *n* (*%*)		94 (9.7)	28 (12.0)	66 (8.9)	.17
Deaths, *n* (*%*)		390 (40.2)	96 (41.2)	294 (39.8)	.71
Age at death, *M* (*SD*)		87.86 (3.48)	88.07 (3.45)	87.80 (3.49)	.58
Follow-up time, *M* (*SD*)		7.36 (2.10)	7.37 (2.15)	7.35 (2.09)	.20

^§^Data is missing for *n* in feelings of worse memory performance than same aged individuals: 1 (0.1%), comorbid conditions: 4 (0.4%), alcohol consumption: 4 (0.4%), apoE4: 36 (3.7%)

^$^based on the Mini Mental State Examination (MMSE) total score

^†^based on the Lawton and Brody scale total score

^‡^based on the Geriatric Depression Scale (GDS) total score

^¥^based on guidelines by the World Health Organization (WHO), no subjects with alcohol dependence

- continuous variables calculated with Mann-Whitney *U* Test

- categorical/dichotomous variables calculated with Chi-Square-Tests

- all tests two-tailed at 95% significance level

### SCD and mortality

The univariate Cox regression analysis did not yield a significant association of SCD and mortality (HR = 1.02, 95%-CI = 0.81–1.28; *p* = .90 in reference to the absence of SCD). In the multivariate Cox regression analysis adjusted for socio-demographic and health characteristic, SCD was also not significantly associated with mortality (HR = .90, 95%-CI = 0.71–1.15; *p* = .40 in reference to the absence of SCD) (Table 2).

**Table 2:** Univariate and multivariate Cox proportional hazards model for the impact of incident subjective cognitive decline (SCD) on mortality (n = 930).

Variables^†^		Hazard ratio	95% confidence interval	*P* value
**UNIVARIATE MODEL**^∑^				
SCD		1.02	0.81–1.28	.90
**MULTIVARIATE MODEL**^£^^,^^ß^				
SCD		0.90	0.71–1.15	.40
**Age, every additional year**		**1.14**	**1.10–1.17**	**< .001**
Male gender		1.16	0.86–1.57	.34
Education				
	High	1^ŧ^		
	Middle	0.94	0.63–1.40	.76
	Low	0.96	0.66–1.39	.82
Marital status				
	Single	1^ŧ^		
	Married/cohabiting	1.14	0.64–2.04	.66
	Divorced	1.11	0.58–2.12	.75
	Widowed	1.43	0.88–2.34	.15
Living situation				
	Private household, alone	1^ŧ^		
	Private household, with relatives	1.33	0.94–1.88	.11
	Residential care	1.34	0.68–2.65	.40
**Cognitive functioning**^**$**^**, every additional point**		**0.91**	**0.85–0.98**	**< .05**
**Impairment in instrumental activities of daily living**^‡^		**1.59**	**1.07–2.37**	**< .05**
Depressive symptoms^§^, every additional point		1.01	0.95–1.07	.81
Comorbid conditions				
	Diabetes mellitus	1.17	0.92–1.49	20
	Hypertension	1.06	0.82–1.36	.67
	**Cardiac arrhythmias**	**1.40**	**1.12–1.76**	**< .01**
	Coronary heart disease	1.01	0.78–1.32	.92
	**Myocardial infarction**	**1.80**	**1.27–2.56**	**< .01**
	Stenosis (of afferent brain vessels)	0.94	0.44–2.02	.88
	Transient ischaemic attack (TIA)	0.91	0.58–1.43	.67
Smoking				
	Non-smoker	1^ŧ^		
	Former smoker	1.26	0.98–1.63	.07
	**Current smoker**	**2.39**	**1.71–3.34**	**< .001**
Alcohol consumption^¥^				
	No drinking	1^ŧ^		
	Normal drinking	1.06	0.84–1.33	.65
	**Risky drinking**	**2.03**	**1.22–3.35**	**< .01**
apoE4				
	No apoE4	1^ŧ^		
	apoE4	0.87	0.65–1.16	.34
**Subsequent incident dementia**		**1.69**	**1.27–2.26**	**< .001**

^†^41 (4.2%) subjects of the initial study sample (*n* = 971) were excluded because of missing values in covariates. Models included 559 survivors and 371 deceased subjects. There was no difference in the proportion of survivors and deceased subjects in the model sample compared to the initial sample (*χ*^*2*^(1, 1901) = 0.10, *p* = .92).

^∑^The proportional hazards assumption was met (*χ*^*2*^(1, 930) = 0.02, *p* = .88).

^£^Age, cognitive functioning (MMSE) and depressive symptoms (GDS) were implemented as continuous variables, all others as categorical

^ß^Method enter was applied, the proportional hazards assumption was met (*χ*^*2*^(26, 930) = 36.28, *p* = .09).

^ŧ^Reference category

^$^based on the Mini Mental State Examination (MMSE) total score

^‡^based on the Lawton and Brody scale total score

^§^based on the total score of the Geriatric Depression Scale (GDS)

^¥^based on guidelines by the World Health Organization (WHO), no subjects with alcohol dependence

The proportional hazards assumption as tested by calculating Schoenfeld residuals was met for the univariate Cox model (*χ*^*2*^(1, 930) = 0.02, *p* = .88) as well as for the multivariate (*χ*^*2*^(26, 930) = 36.28, *p* = .09).

### SCD in relation to concerns and mortality

In the univariate Cox regression analysis, there was also no significant association of mortality and SCD in relation to concerns (HR = 0.95, 95%-CI = 0.57–1.57; *p* = .84; without concerns: HR = 1.03, 95%-CI = 0.80–1.32; *p* = .82 in reference to no SCD). The same was true for the multivariate Cox model on mortality risk which yielded a HR of 0.85 (95%-CI = 0.50–1.45, *p* = .55) for SCD with related concerns and a HR of 0.91 (95%-CI = 0.70–1.18; *p* = .48) for SCD without related concerns in reference to no SCD (Table 3). The proportional hazards assumption as tested by calculating Schoenfeld residuals was also met for the univariate Cox model (*χ*^*2*^(2, 930) = 0.31, *p* = .86) as well as for the multivariate (*χ*^*2*^(27, 930) = 36.69, *p* = .10) when SCD was differentiated in relation to concerns.

**Table 3:** Univariate and multivariate Cox proportional hazards model for the impact of incident subjective cognitive decline (SCD) in relation to concerns on mortality (n = 930).

Variables^†^		Hazard ratio	95% confidence interval	*P* value
**UNIVARIATE MODEL**^∑^				
SCD				
	No	1 ^ŧ^		
	Without related concerns	1.03	0.57–1.57	.82
	With related concerns	.95	0.80–1.32	.84
**MULTIVARIATE MODEL**^£^				
SCD				
	No	1 ^ŧ^		
	Without related concerns	0.91	0.70–1.18	.48
	With related concerns	0.85	0.50–1.45	.55
**Age, every additional year**		**1.14**	**1.10–1.17**	**< .001**
Male gender		1.16	0.86–1.56	.34
Education				
	High	1^ŧ^		
	Middle	0.96	0.66–1.39	.82
	Low	0.94	0.63–1.40	.76
Marital status				
	Single	1^ŧ^		
	Married/cohabiting	1.13	0.63–2.03	.67
	Divorced	1.11	0.58–2.11	.76
	Widowed	1.43	0.87–2.34	.16
Living situation				
	Private household, alone	1^ŧ^		
	Private household, with relatives	1.33	0.94–1.88	.11
	Residential care	1.35	0.68–2.67	.39
**Cognitive functioning**^**$**^**, every additional point**		**0.91**	**0.85–0.98**	**< .05**
**Impairment in instrumental activities of daily living**^‡^		**1.59**	**1.07–2.37**	**< .05**
Depressive symptoms^§^, every additional point		1.01	0.95–1.07	.79
	Diabetes mellitus	1.17	0.92–1.49	.21
	Hypertension	1.05	0.82–1.35	.68
	**Cardiac arrhythmias**	**1.40**	**1.12–1.76**	**< .01**
	Coronary heart disease	1.02	0.78–1.32	.91
	**Myocardial infarction**	**1.80**	**1.27–2.56**	**< .01**
	Stenosis (of afferent brain vessels)	0.95	0.44–2.04	.90
	Transient ischaemic attack (TIA)	0.90	0.57–1.42	.66
Smoking				
	Non-smoker	1^ŧ^		
	Former smoker	1.26	0.98–1.63	.07
	**Current smoker**	**2.39**	**1.71–3.33**	**< .001**
Alcohol consumption^¥^				
	No drinking	1^ŧ^		
	Normal drinking	1.06	0.84–1.33	.64
	**Risky drinking**	**2.03**	**1.23–3.36**	**< .01**
apoE4				
	No apoE4	1^ŧ^		
	apoE4	0.87	0.65–1.16	.34
**Subsequent incident dementia**		**1.69**	**1.27–2.26**	**< .001**

^†^41 (4.2%) subjects of the initial study sample (*n* = 971) were excluded because of missing values. Models included 559 survivors and 371 deceased subjects. There was no difference in the proportion of survivors and deceased subjects in the model sample compared to the initial sample (*χ*^*2*^(1, 1901) = 0.10, *p* = .92).

^∑^The proportional hazards assumption was met (*χ*^*2*^(2, 930) = 0.31, *p* = .86).

^£^Method enter was applied, the proportional hazards assumption was met (*χ*^*2*^(27, 930) = 36.69, *p* = .10); age, cognitive functioning (MMSE) and depressive symptoms (GDS) were implemented as continuous variables, all others as categorical variables.

^ŧ^Reference category

^$^based on the Mini Mental State Examination (MMSE) total score

^‡^based on the Lawton and Brody scale total score

^§^based on the total score of the Geriatric Depression Scale (GDS)

^¥^based on guidelines by the World Health Organization (WHO), no subjects with alcohol dependence

The results of both multivariable Cox proportional hazard regression models to assess the association of SCD and mortality were based on 930 cases of the original study sample (*n* = 971) due to missing values in covariates (*n* = 41, 4.2%). However, the proportion of deceased subjects and survivors did not differ between the two samples (*χ*^*2*^(1, 1901) = 0.10, *p* = .92). Also, sample reduction did not cause differences in any other considered variable used in the Cox regression compared to the initial sample (results not shown).

### Sensitivity analyses

To avoid potential over-correction in the adjustment of the multivariate Cox models, we conducted a sensitivity analysis for the association of SCD as well as SCD in relation to concerns, respectively, and mortality without considering cognitive functioning (results on the MMSE) and subsequent incident dementia as confounders as the initial study sample was without cognitive impairment per definition. Again, SCD was not associated with mortality (HR = 0.95, 95%-CI = 0-75-1.21; *p* = .68; test of proportional hazards assumption: *χ*^*2*^(24, 930) = 31.99, *p* = .13), neither was SCD in relation to concerns (HR = 0.87, 95%-CI = 0.51–1.49; *p* = .62) or SCD without related concerns (HR = 0.97, 95%-CI = 0.75–1.25; *p* = .79) in reference to no SCD (test of proportional hazards assumption: *χ*^*2*^(25, 930) = 32.41, *p* = .15) (results not further shown).

### Survival times and case-fatality rates

Kaplan Meier analysis identified an overall mean survival time of 8.1 years (*SD* = 0.1). Individuals with incident SCD did not show a significantly different survival time than individuals without SCD (*M* = 8.0, *SD* = 0.1 vs. *M* = 8.1, *SD* = 0.10; Log rank test: *p* = .90). The mean survival time for individuals with SCD in relation to concerns was 7.9 years (*SD* = 0.4) and *M* = 8.0 years (*SD* = 0.2) without related concerns compared to no SCD (*M* = 8.1, *SD* = 0.1; Log rank test: *p* = .95). The median was not reached yet.

In terms of case-fatality rates, overall mortality resulted in 54.6 cases (95%-CI = 48.3–60.7) per 1,000 person-years. In individuals with SCD, case-fatality cumulated in a rate of 55.9 (95%-CI = 45.8–68.3) and in individuals without SCD in 54.2 (95%-CI = 48.3–60.7). When SCD was related to concerns, the case-fatality rate was 52.4 (95%-CI = 32.1–85.6), without related concerns it was 56.7 (95%-CI = 45.5–70.5) and in the absence of SCD 54.2 (95%-CI = 48.3–60.7).

## Discussion

We aimed to investigate the association of subjective cognitive decline (SCD) in memory and mortality in incident cases in the elderly. After observing for a follow-up time of 7.5 years, we did not find incident SCD being associated with increased mortality adjusted for potentially confounding socio-demographic covariates, comorbid conditions and subsequent dementia in individuals aged 81 years on average. The same was true if we differentiated SCD in relation to concerns. Importantly, there was no relation to mortality even though subjects with SCD had more depressive symptoms and more frequently suffered a TIA than subjects without SCD–both conditions are themselves associated with increased mortality (e.g. [43,44]).

Our results from incident SCD cases are thus in line with most of the previous studies that examined the association between SCD in memory performance and mortality in prevalent cases [19–25]. To our knowledge, only one study reported overall evidence for an association between SCD and increased mortality [26]. Besides that, Ogata et al. [27] suggested a possible increased one-year-mortality-rate in women with SCD, but not in men.

However, relatedness between these studies’ results has to be seen with caution due to a significant variance in populations and methods. For example, studied age differed from 58 [25] to 82 [26] years on average, follow-up times ranged from three [22] to ten years [25] and study samples varied from representative samples (e.g. [24]) to selected samples like nursing-home residents [22]. Besides that, the operationalization of SCD was very heterogeneous. For example, studies assessed SCD either as the frequency of the occurrence of memory problems [24], as part of a more global measure of self-reported health [21,24], as memory loss [20], as a feature of subjective cognitive functioning next to confusion and recognizing problems [23] or as a measure of subjective cognitive complaints [25]. Assessment of SCD varied from asking single questions (e.g. [23,24,19,21,26]) to applying standardized batteries [25]. Future studies may investigate more specifically defined cases of cognitive complaints, such as in relation to future cognitive decline or depression. Concerning SCD as potentially earliest symptomatic manifestation of AD, a conceptual framework has been recently proposed providing consensus criteria for research [4]. According to this, SCD is not only restricted to memory performance, as investigated in this study as one type of SCD, even though the association of memory function with preclinical AD may be strongest at present. Jessen et al. [4] suggested that SCD also comprises subjectively experienced worsening of capacities among other cognitive domains besides memory which is reasonable since a) the first cognitive symptoms of AD are not limited to memory decline and b) subjects may report memory decline when they actually experience a decline in a different cognitive domain, e.g. executive function, and vice versa. An investigation of such other domains of SCD besides the memory type in regard to mortality might be useful. This is supported by the fact that some of the prevalence studies reported increased mortality in other cognitive domains but memory. For example, reports on the frequency of the occurrence of confusion were related to an increased mortality in a study with 7,527 subjects aged over 70 years who were followed-up for 7 years [24]. Specific cognitive symptoms like difficulty in mental calculation (HR = 1.3) as well as medical advice seeking due to cognitive symptoms (HR = 1.4) were associated with a higher mortality risk in 15,510 subjects aged 58 years on average [25]. And, finally, reports of problems in recognizing familiar people served as a predictor of all-cause mortality in a study of 4,921 subjects aged over 60 years followed-up for 7 years [23]. Spoken in terms of the newly proposed SCD concept, subjectively perceived memory decline as one special type of SCD might not impact mortality, but maybe other cognitive features of the non-memory domains inherent in SCD do. This may be subject to incidence studies.

Since the majority of the varying studies could not find evidence for an association of SCD in memory performance with mortality in prevalent and incident cases, such an association might ultimately not apply. One explanation might be that SCD does not ultimately lead into future cognitive decline in any case. Though conversion rates to MCI and dementia are indeed higher in individuals with SCD, namely about twice as high compared to no SCD, it is about one quarter (26.6%) of individuals with SCD who subsequently develop MCI and about 14.1% who progress to dementia, as a meta-analysis revealed [45].

Besides that, memory complaints are a broad phenomenon which could also be related to normal ageing, psychiatric, neurological and medical diseases other than dementia (e.g. major depression and anxiety), substance abuse, personality traits or medication intake [4]. Moreover, as Lahr et al. [46] stated, complaints about memory problems may additionally reflect general levels of low mood or physical health.

Some limitations of this study have to be addressed. First, the sample might lack representativity, even though more than 91% of the German elderly population regularly consults a GP [47]. The generalizability of our results may be limited due to a moderate response rate with a significant number of GP patients who refused study participation or who could not be contacted. Non-respondents might have presented a different distribution of cognitive functioning.

Second, we assigned the onset of SCD by convention to the midpoint between two measurement waves, whereby the latter constitutes the point of diagnosis. On average, the SCD onset can be assumed at this midpoint. However, this method may be associated with some inaccuracy.

Third, SCD was assessed by asking two simple questions about memory and related concerns. A more comprehensive questionnaire might have revealed more differentiated results, especially in regard to non-memory domains of SCD. Until now there is a lack of a gold standard on how to assess SCD, but currently first preliminary recommendations for future research were proposed [48].

## Conclusion

We suggest that incident SCD in memory may not be associated with increased mortality in the elderly. This might be mainly due to the fact that SCD does not necessarily lead into future cognitive decline in any case. However, as some prevalence studies imply, subjectively perceived decline in other non-memory cognitive domains could be associated with an increased mortality risk. This might be investigated in future incidence studies.
